# Gender differences in the association between anxiety and behavioral problems in 31,400 Chinese children: a cross-sectional regression and network analysis

**DOI:** 10.1186/s12888-026-08371-9

**Published:** 2026-07-14

**Authors:** Shurui Chen, Zheng Zhang, Zhen Tao, Jingyan Sun, Xiaoping Wang, Jiansong Zhou

**Affiliations:** 1 Department of Psychiatry, National Clinical Research Center for Mental Disorders, and National Center for Mental Disorders, The Second Xiangya Hospital of Central South University, Changsha, Hunan China; 2 Key Laboratory of Brain, Cognition and Education Sciences, Ministry of Education, Guangzhou, China; 3 School of Psychology, Center for Studies of Psychological Application, Guangdong Key Laboratory of Mental Health and Cognitive Science, South China Normal University, Guangzhou, China; 4 Liuyang Brain Hospital, Liuyang, Hunan China

**Keywords:** Anxiety, Behavioral problems, Gender differences, Psychopathology

## Abstract

**Background:**

Child anxiety and behavioral problems are significant global public health concerns. Traditional methods often fail to capture intricate symptom interactions. This study used network analysis to explore the relationship between anxiety and behavioral problems, focusing on gender differences in a large sample of Chinese children.

**Methods:**

Data from 31,400 students (aged 9–16) were analyzed. Anxiety and behavioral problems were assessed using SCARED and PSQ. To control for developmental variance, symptom domains were residualized against chronological age prior to network estimation.

**Results:**

Girls reported higher anxiety, while boys showed more behavioral problems. Generalized anxiety demonstrated the strongest independent association with behavioral problems, closely followed by school avoidance. Network analysis revealed significant gender differences in structure (M = 0.277, *p* < 0.001) and global strength (S = 0.303, *p* < 0.001). Boys exhibited higher global strength (Boys = 5.881, Girls = 5.578). Centrality stability was high (CS = 0.75), with Hyperkinesis and Generalized Anxiety acting as core domains for both genders.

**Conclusions:**

Child psychopathology features distinct gender-specific network structures independent of age. These findings provide preliminary insights into the domain-level architecture of child psychopathology, which may inform the development of gender-sensitive screening and supportive strategies in school settings.

**Trial registration:**

Clinical trial number: not applicable.

**Supplementary Information:**

The online version contains supplementary material available at https://doi.org/10.1186/s12888-026-08371-9.

## Introduction

Child anxiety disorders and behavioral problems are two of the most prevalent forms of affective and emotional disorders in youth, with profound, complex impact on child development, families, and society [1, 2]. Traditionally viewed as distinct internalizing and externalizing disorders, recent evidence suggests they may share underlying pathological mechanisms, falling along a broader affective disorder spectrum [3, 4]. Understanding the intricate relationships between these domains is crucial for early risk identification within school settings.

Anxiety stands as a significant contributing factor to child behavioral problems [5, 6]. The underlying mechanisms linking internalizing anxiety to externalizing behavioral problems are hypothesized to involve intricate cognitive, neurobiological, and socio-environmental processes. Extrapolating from translational neuroscience literature, severe emotional distress may potentially compromise executive functioning and top-down cognitive control, theoretically driven by alterations in critical neural networks—such as those linking the prefrontal cortex to the ventral tegmental area—which are speculated to regulate behavioral control and emotional reactivity [7]. Furthermore, persistent anxiety frequently destabilizes school functioning and peer relationships, establishing a distressing feedback loop where academic avoidance and social friction can fuel impulsive or defiant behavioral outbursts [8]. Because internalizing distress is often highly covert, while behavioral disruptions are overtly visible, utilizing a single-informant design can severely bias the observed comorbidity architecture. Consequently, integrating child self-reports (capturing subjective emotional states) with parent observations (capturing observable behavioral manifestations) provides a methodologically rigorous, cross-informant assessment capable of disentangling the true interplay between these co-occurring developmental domains. However, these sub-clinical, domain-level interactions do not operate in a vacuum; they are heavily filtered by gender-specific socialization and societal expectations [9, 10]. From a socio-constructivist perspective, children are socialized early into gender-congruent roles where boys are frequently encouraged to adhere to norms of ‘toughness’ and emotional stoicism, while girls are granted more latitude to express vulnerability and fear [10]. Consequently, behavioral disruptions in males may often act as an externalizing proxy for underlying, suppressed emotional turmoil, whereas gender norms in females may more directly amplify internalizing pathways [9, 11, 12]. These intricate, multi-layered mechanisms underscore the complex dimensional interplay within the broader youth psychopathology spectrum.

While traditional methods, such as regression analysis, have demonstrated significant associations between these symptom domains, they often fail to capture the intricate, domain-level interactions that may drive the persistence and comorbidity of affective disorders. Network analysis offers a powerful alternative by modeling symptoms as nodes and their direct statistical relationships as edges, providing a unique window into the structural architecture of psychopathology [13, 14]. Recent network research has begun to clarify gender-specific patterns in youth psychopathology, offering critical baseline comparisons. For instance, Abend et al. [15] demonstrated the cross-gender and cross-age stability of generalized anxiety symptoms as prominent network cores in a large cohort [15]. Similarly, Angulo-Salas et al. [16] mapped adolescent symptom networks, revealing that the relational configuration among resilience, happiness, and mental health (anxiety, depression, and stress) is largely comparable across sex [16]. Extending network boundaries to school environments, Zdoupas [17] established how school-related well-being and academic anxiety tightly integrate into adolescent psychological architectures [17]. Despite these insights, points of divergence emerge: prior studies either focused exclusively on internalizing structures or treated school functioning as an isolated outcome. Furthermore, because multi-informant inventories inherently involve conceptual overlap—such as the parent-rated Conners scale capturing internalizing elements like anxiety and psychosomatic problems—the estimated network architecture potentially conflates different dynamics. Certain cross-scale edges may represent cross-informant convergent validity regarding a child’s internalizing distress, while other connections map genuine cross-domain bridging pathways that link emotional vulnerabilities directly to externalizing behavioral disruptions. The specific, multi-informant mechanisms by which individual anxiety domains bridge to distinct behavioral difficulties remain underexplored. The present study addresses this gap by mapping the explicit internalizing-externalizing bridging pathways while clearly differentiating cross-informant construct convergence, within a population of 31,400 Chinese children, where unique socio-cultural stressors may shape distinct cross-domain architectures.

To address these gaps, the present study proposes three testable hypotheses: (H1) GA and Hyperkinesis will serve as core domains regardless of demographic sex; (H2) boys and girls will exhibit significant structural and global strength differences in their anxiety-behavioral problem networks, with distinct topological features driving their respective psychopathological architectures; and (H3) school avoidance will function as a significant bridge node linking emotional distress to behavioral outcomes. Concurrently, the substantive contribution of the present study transcends mere population-based demographic documentation, operating instead at three distinct levels: methodologically, it establishes a cross-informant linkage framework—coupling child self-reported anxiety with parent-observed behavioral patterns—to minimize common-method variance; topologically, it provides a comprehensive subscale-level network comparison to delineate the precise structural organization of co-occurring internalizing and externalizing problems; and pragmatically, it suggests age-independent screening indicators optimized for early mental health screening within large-scale, school-based epidemiologic settings.

## Method

### Participants

The study employed a cluster sampling method, targeting fourth-, fifth-, and sixth-grade primary school students and their parents in Liuyang City, Hunan Province. Students meeting the following criteria were invited to complete the questionnaire: (1) enrolled in grades 4–6, and (2) able to fully comprehend the questionnaire content. To ensure comprehension, particularly for younger students (ages 9–10), classroom teachers and trained research assistants provided a standardized explanation of the items before the survey began, and participants were encouraged to seek clarification for any difficult vocabulary during the session. The exclusion criteria were as follows: (1) refusal to participate in the survey. Data collection was coordinated by the Liuyang Education Bureau in 2023. In accordance with the Ministry of Education of the People’s Republic of China’s guidelines on strengthening mental health monitoring for primary and secondary school students, the Liuyang Education Bureau distributed informed consent forms to parents collectively. Data was not collected for students whose parents did not provide consent. Similarly, during data collection, classroom teachers obtained students’ verbal consent on-site; those who declined participation were excluded. A total of 32,043 questionnaires were received, of which 615 were excluded following a strict listwise deletion protocol (i.e., any questionnaire with one or more missing responses in either the SCARED or PSQ scales was discarded) and 28 were excluded due to incomplete or erroneous personal information. This resulted in 31,400 valid questionnaires. Demographic information, including age, sex (categorized as boys or girls), and grade, was retrieved directly from the official school registration databases provided. This study is part of the China Depression Cohort Study (CDCS) and was approved by the Ethics Committee of the Second Xiangya Hospital, Central South University (LYF2022031).

### Screen for child anxiety related emotional disorders (SCARED)

The Screen for Child Anxiety Related Emotional Disorders (SCARED) is a self-report questionnaire consisting of 41 items designed to screen for anxiety disorders in children [18, 19]. It includes five subscales: Panic Disorder or Significant Somatic Symptoms (items 1, 6, 9, 12, 15, 18, 19, 22, 24, 27, 30, 34, 38), Generalized Anxiety (items 5, 7, 14, 21, 23, 28, 33, 35, 37), Separation Anxiety (items 4, 8, 13, 16, 20, 25, 29, 31), Social Anxiety (items 3, 10, 26, 32, 39, 40, 41), and School Avoidance (items 2, 11, 17, 36). Participants rate each item on a 3-point scale (0 = almost never, 1 = sometimes, 2 = often). The total score is the sum of the 41 item scores, ranging from 0 to 82. A total score of 25 or higher suggests a potential anxiety disorders, with scores above 30 indicating a higher likelihood. The score for each subscale is the sum of the items within that subscale. In this study, the Cronbach’s alpha for the scale was 0.931. The Cronbach’s alpha for the five subscales were as follows: Panic Disorder or Significant Somatic Symptoms (0.826), Generalized Anxiety (0.826), Separation Anxiety (0.754), Social Anxiety (0.804), and School Avoidance (0.600). While the School Avoidance subscale was below the 0.70 threshold, this is expected given its short length (4 items), and it remains within the acceptable range for research purposes.

### Conners parents symptom questionnaire (PSQ)

The Conners Parent Symptom Questionnaire (PSQ) was developed by Conners in 1969 and later revised by Goytle, Conners, and Ulrich in 1978 [20]. It is completed by a child’s father or mother and is used to screen for behavioral problems in children [20]. The PSQ consists of 48 items, grouped into six subscales: Conduct Problems (items 2, 8, 14, 19, 20, 21, 22, 23, 27, 33, 34, 39), Learning Problems (items 10, 25, 31, 37), Psychosomatic problem (items 32, 41, 43, 44, 48), Impulsive Hyperactivity (items 4, 5, 11, 13), Anxiety (items 12, 16, 24, 47), and Hyperactivity Index (items 4, 7, 11, 13, 14, 25, 31, 33, 37, 38). Each item is rated on a 4-point scale, from 0 (never) to 3 (very often). The scores for each subscale are obtained by summing the item scores, with higher scores indicating more severe behavioral problems. A score on any subscale that exceeds the average score for same-age, same-gender children by 2 standard deviations is considered indicative of a behavioral problem [21]. The Chinese version of the PSQ has demonstrated good reliability and validity [22]. In this study, the Cronbach’s alpha for the scale was 0.948. The subscale-level alphas ranged from 0.599 to 0.874 (Conduct Problems: 0.874; Learning Problems: 0.742; Impulsive Hyperactivity: 0.644; Anxiety: 0.599; Hyperactivity Index:0.837). Consistent with prior literature, lower alpha values in certain subscales are likely due to the limited number of items (e.g., 4 items per subscale) rather than a lack of internal coherence.

### Measurement invariance testing

To ensure that the subsequent comparisons of SCARED and PSQ scores across sexes were methodologically sound, multi-group confirmatory factor analyses (MG-CFA) were performed using the lavaan and semTools packages in R 4.3.3. Because our network and invariance models were operationally specified at the aggregate subscale level rather than the individual item level, the composite domain scores were treated as continuous variables. To robustly account for multi-variate non-normality and potential distribution skewness inherent in a large community screening cohort (*N* = 31400), the Robust Maximum Likelihood (MLR) estimator with Satorra-Bentler scaling corrections was employed.

Measurement invariance was evaluated using a progressively constrained nested model hierarchy: configural invariance (unconstrained factor structures), metric invariance (cross-sex equality constraints on factor loadings), and scalar invariance (simultaneous cross-sex equality constraints on factor loadings and item intercepts). Following established psychometric guidelines [23, 24], because the Satorra-Bentler χ^2^ difference test is overly sensitive to large sample sizes, model equivalence was deemed established if the change in the robust Comparative Fit Index (ΔCFI) was ≤ 0.010 and the change in the robust Root Mean Square Error of Approximation (ΔRMSEA) was ≤ 0.015. Absolute fit was monitored via scaling-corrected χ^2^, degrees of freedom (df), CFI, RMSEA, and Standardized Root Mean Square Residual (SRMR).

### Statistical analyses

Cronbach’s alpha for the two scales was calculated using JASP 0.16.2 [25]. Data processing, including missing value deletion, variable assignment, and factor calculation for the scales, was conducted using SPSS 22.0, along with descriptive statistics and z-tests to identify any significant differences between variables. A Pearson partial correlation analysis, controlling for gender and age, was conducted to examine the associations between the factor scores of the Screen for Child Anxiety Related Emotional Disorders (SCARED) and the Problem Behavior Questionnaire (PSQ). The results were visualized using JASP (version 0.16.2) [25]. Standardized binary and multivariate logistic regression configurations were implemented to quantify the independent predictive value of specific anxiety dimensions on behavioral disruptions. To facilitate unbiased, direct effect-size comparisons across subscales of varying lengths and variances, all five SCARED predictors were transformed into standardized Z-scores prior to model entry. Multicollinearity diagnostics were systematically conducted by examining Tolerance and Variance Inflation Factors (VIF) across all regression blocks to ensure the mathematical stability of these multivariate parameters. Finally, the network structure of anxiety and behavioral problems in boys and girls was estimated using R 4.3.3, including network description (such as node centrality and network comparison), and network stability analysis [26].

### Network estimation and visualization

The Gaussian graphical model (GGM) was applied to estimate the networks for the four samples [27]. To account for potential confounding effects of age given the wide age range (9–16 years) of our cohort, all symptom domains were residualized against chronological age prior to network estimation. Specifically, we conducted linear regressions for each node with age as the independent variable and used the resulting residuals for network construction. This ensures that the estimated edge weights represent associations independent of age-related developmental variance. This procedure was applied separately to the male and female sub-samples before conducting gender comparisons. Network visualization was performed using the R packages ‘bootnet’ and ‘qgraph’, where ‘nodes’ represent the subscale scores of the SCARED and PSQ scales, and ‘edges’ represent the regularized partial correlations between the ‘nodes’ [28]. Within the parent-report PSQ instrument, certain adjacent edges (particularly LP-Hyper and IH-Hyper) must be interpreted with appropriate caution, as they partially reflect structural item overlap built into the scale’s design rather than purely independent psychopathological comorbidity. This level of granularity was selected because subscales represent validated, clinically recognizable symptom domains, providing a more parsimonious and stable architecture for understanding the interplay between complex internalizing and externalizing constructs than item-level analysis. Given that these nodes capture dimensional psychological constructs via standardized screening scales in a general population, they are conceptually operationalized as trait-level dimensional domains (or symptom domains) rather than clinically assessed individual symptoms. For narrative fluidity and alignment with psychopathological network nomenclature, these aggregate constructs are referred to as “domains” or “symptom clusters” at the node level throughout the network analysis. Consequently, highly central nodes should be interpreted as influential screening domains rather than individualized core symptoms. Furthermore, given the multi-informant architecture of our design, edges estimated within the self-report scale (SCARED), within the parent-report scale (PSQ), and between the two informants are not directly equivalent. The cross-scale connections inherently capture both genuine psychopathological coupling and informant-source variance, reflecting differences in structural reporting criteria. In this model, blue edges represent positive associations and red edges represent negative associations, with thicker lines indicating stronger correlations. Specifically, network edges were estimated using the graphical LASSO (least absolute shrinkage and selection operator) algorithm, with the tuning parameter selected by minimizing the Extended Bayesian Information Criterion (EBIC). In accordance with standard practice for optimizing network sparsity and controlling false-positive rates, the EBIC tuning parameter was set to 0.5.

### Network centrality

We classified domains based on their connectivity within a disorder and across disorders. Core domains are those that exhibit strong interrelationships with other domains within the same disorder, whereas bridge domains are those that link domains from two distinct psychiatric conditions [29–31]. To determine which domains are core domains, we assessed the centrality of nodes. The centrality measures for nodes were calculated using the ‘qgraph’ R package [32]. Additionally, to identify bridge domains, we employed the ‘networkTools’ R package to compute bridge centrality [32]. The most frequently applied centrality metrics include strength, closeness, and betweenness [31]. For bridge domains, the corresponding measures are bridge strength, bridge closeness, and bridge betweenness [31]. However, closeness and betweenness were deemed less reliable and indirect in this context, which makes them unsuitable for symptom networks in mental health research [30]. In complex networks that contain both positive edges (representing “mutual activation” or “positive correlation”) and negative edges (representing “antagonism” or “negative correlation”), traditional Strength Centrality only calculates the sum of absolute edge weights [32]. Consequently, it fails to accurately reflect the cumulative causal effect—whether activation or inhibition—that a node exerts on the overall network. To address this limitation, Robinaugh et al. [33] introduced the Expected Influence (EI) index [33]. Unlike strength, EI is calculated as the algebraic sum of all edge weights connected to a node, allowing for the cancellation of opposite-signed edges. Accordingly, the present study adopted EI and bridge expected influence as the primary metrics to identify core domains and bridge domains, respectively. Domains with the highest EI values were identified as core domains, while those within the top 20% of bridge expected influence were considered bridge domains [31]. These measures are particularly valuable in identifying domains that are most influential in maintaining the disorder or linking different symptom clusters, thereby pinpointing optimal targets for gender-sensitive early risk identification and influential epidemiological screening indicators.

### Network accuracy and stability estimation

Post-hoc stability analyses were performed to evaluate the robustness and precision of edge weights and centrality measures using the ‘bootnet’ R package [32]. First, we assessed the reliability of edge weights by calculating their 95% confidence intervals (CIs) through nonparametric bootstrapping, with 1000 bootstrap samples. Next, following the methodology outlined by Epskamp et al., we evaluated the stability of node centrality by computing the correlation stability (CS) coefficient based on a case-dropping procedure, also using 1000 bootstrap samples [32]. For a centrality index to be considered stable, it should exhibit minimal variation across different subsets. Stability was quantified with CS coefficients, with values above 0.5 considered stable, and values below 0.25 indicating instability [32].

### Network comparison

We used the ‘Network Comparison Test’ (NCT) R package to compare network models of anxious, problem behavior in boys and girls. The comparison was based on 1000 iterations of a permutation procedure [34]. NCT evaluated the differences between the networks from two perspectives: (i) global strength, which refers to the total sum of edge weights within each network, and (ii) structural invariance, which assesses significant variations in the relationships between nodes and variables [35, 36]. Meanwhile, differences of each specific edge were also separately measured. The anxious-problem behavior networks in boys and girls were built and compared using the bootstrap method at a significance level of 0.05.

## Results

### Demographic characteristics

A total of 31,400 children participated in this study, including 16,246 boys and 15,154 girls, aged between 9 and 16 years, and enrolled in grades 4, 5, or 6. Table 1 presents the descriptive statistics of The Screen for Child Anxiety Related Emotional Disorders (SCARED) and the Conners Parent Symptom Questionnaire (PSQ), as well as the results of U-tests comparing boys and girls.

**Table 1:** Descriptive statistics of the boys and girls groups, including the Z-value and p-value from the U-test comparing the two groups (*n* = 31400)

Labels or variables	Symptoms	Boys(*n* = 16246)		Girls(*n* = 15154)		Z	*p*	Cohen’s d
		Mean	SD	Mean	SD			
PD	Panic Disorder or Significant Somatic Symptoms	2.25	2.90	2.42	3.16	-3.316	0.001	0.019
GA	Generalized Anxiety Disorder	2.03	2.60	2.48	3.01	-11.886	< 0.001	0.067
SepA	Separation Anxiety Disorder	2.06	2.46	2.28	2.60	-7.295	< 0.001	0.041
SoA	Social Anxiety Disorder	2.92	2.76	3.51	2.96	-18.136	< 0.001	0.102
SA	School Avoidance	0.80	1.13	0.93	1.24	-8.664	< 0.001	0.049
CP	Conduct problem	3.84	4.48	3.52	4.31	-2.900	0.004	0.016
LP	Learning problem	2.18	2.27	1.87	2.08	-11.399	< 0.001	0.064
Psy	Psychosomatic	0.79	1.50	0.93	1.66	-3.647	< 0.001	0.021
IH	Impulsive-hyperactive	1.61	1.80	1.49	1.74	-4.062	< 0.001	0.023
Anx	Anxiety	0.93	1.38	1.05	1.45	-4.790	< 0.001	0.027
Hyper	Hyperkinesis Index	4.12	4.14	3.60	3.82	-7.123	< 0.001	0.040

Significant differences were observed between boys and girls across all dimensions of the SCARED and PSQ. However, given the statistical power of the extremely large sample size (*N* = 31,400), these differences should be interpreted with appropriate caution; calculation of standardized mean differences revealed that the absolute effect sizes for all subscale domains were small to trivial (Cohen’s d ranging from 0.016 to 0.102, see Table 1), suggesting that the practical significance of these population-level mean deviations is modest.

Regarding the positive rates of the scales, the analysis revealed that the positive rates for the SCARED and PSQ scales in all subjects were 10.20% and 13.99%, respectively. The positive rate of the SCARED scale in girls (11.88%) was significantly higher than in boys (8.64%) (X2 = 90.310, *p* < 0.001), while the positive rate of the PSQ scale in boys (14.47%) was significantly higher than in girls (13.48%) (X2 = 6.303, *p* = 0.012).

### Measurement invariance across genders

To confirm the structural equivalence of the assessment tools across groups, a series of progressively constrained multi-group CFAs were performed for the SCARED and PSQ scales(Table S1). For the SCARED scale, the configural model demonstrated an acceptable fit to the data (RMSEA = 0.046, SRMR = 0.043, CFI = 0.868). The subsequent imposition of equality constraints on factor loadings (metric invariance) and item intercepts (scalar invariance) did not result in a significant decrement in model fit, as evidenced by the minimal changes in CFI and RMSEA (ΔCFI = -0.001 and ΔRMSEA = 0 for the metric model; ΔCFI = -0.003 and ΔRMSEA = 0 for the scalar model). However, it should be noted that while the nested model comparisons supported progressive invariance, the baseline configural model for the SCARED scale yielded a suboptimal absolute fit (CFI = 0.868). Consequently, subsequent mean-level and structural network comparisons between genders should be interpreted with appropriate psychometric caution.

Similarly, for the PSQ scale, strong measurement invariance was established across genders. The transition from the configural model (RMSEA = 0.051, SRMR = 0.043) to the metric model yielded a ΔCFI of -0.003 and a ΔRMSEA of 0, while the further transition to the scalar model resulted in a ΔCFI of -0.004 and a ΔRMSEA of 0. These results rigorously justify the validity of the subsequent mean-level and network structure comparisons between boys and girls.

### Correlation analysis

**Fig. 1:**
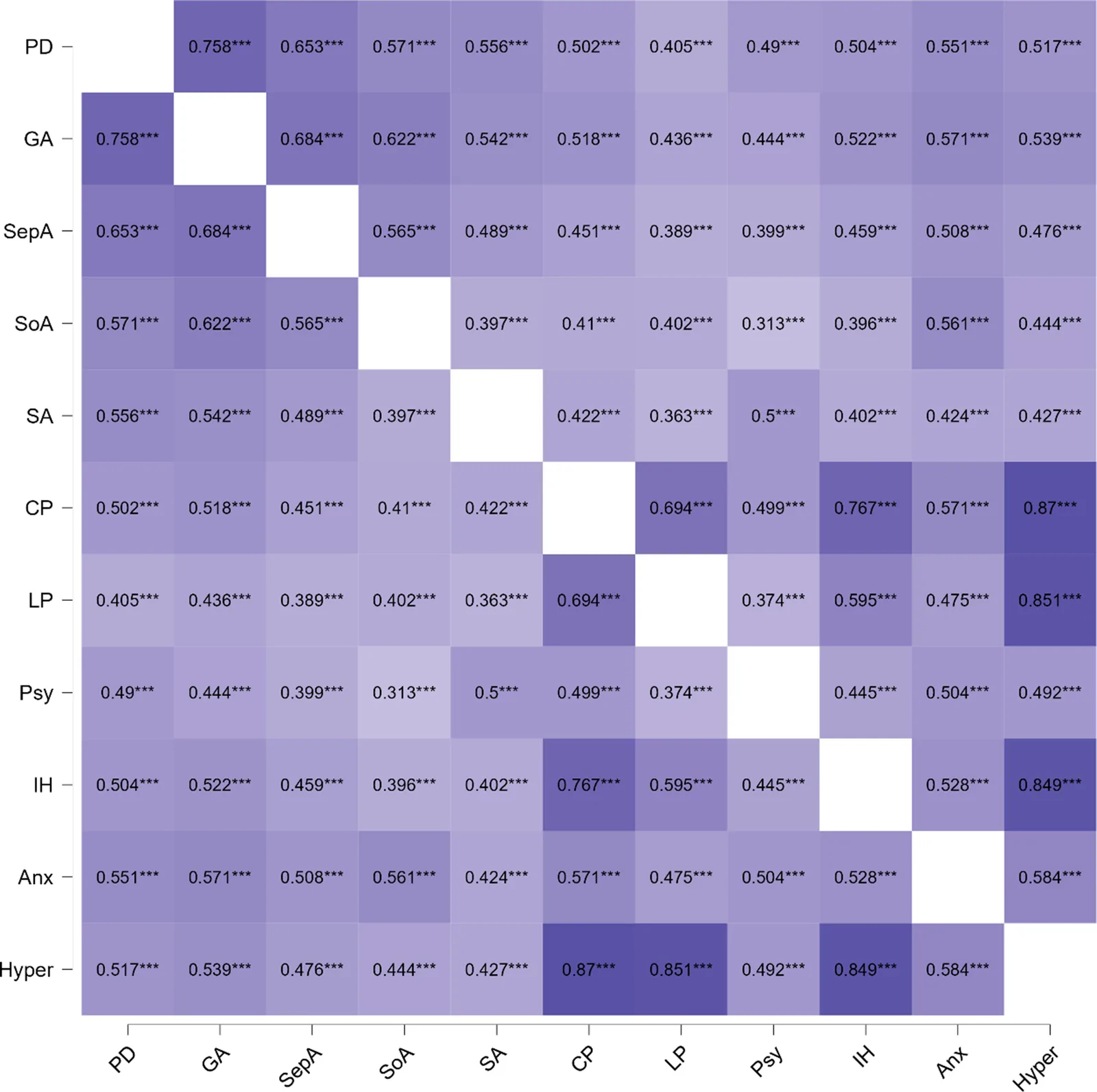
Partial Pearson’s r heat map of the correlation between symptoms of anxiety and behavioral problems. Notes: The intensity of the shaded purple tiles reflects the magnitude of the partial correlation coefficient (r), with darker cells indicating stronger co-variance independent of age and sex covariates. ∗∗∗: *p* < 0.001

In the correlation analysis, gender and age were used as control variables, and the factor scores of the SCARED and PSQ scales were included in the statistical analysis, generating a partial correlation heatmap (Fig. 1). A significant positive correlation was found between anxiety symptoms and behavioral problems.

### Regression analysis

This study employed binary logistic regression and multiple logistic regression analyses to explore the associations between total anxiety scores and different types of anxiety on behavioral problems. After controlling for age, total anxiety scores significantly associated with the presence of behavioral problems (Table 2). The odds ratio (OR) for boys was 1.125 (*p* < 0.001), and for girls, it was 1.131 (*p* < 0.001). This indicates that for every 1-point increase in total anxiety scores, the likelihood of behavioral problems increased by 12.5% in boys and 13.1% in girls. After controlling for total anxiety scores, age had no significant independent association with behavioral problems (*p* > 0.05), suggesting that the statistical link between total anxiety scores and behavioral problems was independent of age.

**Table 2:** Binary logistic regression analysis results for boys and girls

Sex	Variable	B	Standard Error	Wald	df	*p*	Exp(B)
Boy	Age(years)	0.026	0.038	0.452	1	0.501	1.026
	SCARED Total	0.118	0.003	2143.486	1	<0.001	1.125
	Constant	-3.694	0.415	79.093	1	<0.001	0.025
Girl	Age(years)	0.020	0.044	0.210	1	0.646	1.020
	SCARED Total	0.123	0.003	2228.870	1	<0.001	1.131
	Constant	-4.115	0.469	76.851	1	<0.001	0.016

Notes: Dependent Variable: Problem behavior status (coded as 1 for presence and 0 for absence), defined as exceeding the age- and sex-specific community norms by 2 standard deviations (+ 2 SD) on one or more of the PSQ subscales or the Hyperkinesis Index. SCARED Total: Sum of scores from the Screening for Child Anxiety-Related Emotional Disorders (SCARED) scale

Among specific domains of anxiety, Z-score standardized multivariate logistic regression configurations revealed a refined hierarchy of effect sizes across both sexes, evaluated via standardized odds ratios per standard deviation change (OR). After neutralizing scale length and variance artifacts through standardization, generalized anxiety (GA) emerged as the single strongest independent correlate of behavioral problem status across both sexes (Boys: OR = 1.498, 95% CI: 1.393–1.611, Wald = 118.524; Girls: OR = 1.477, 95% CI: 1.371–1.591, Wald = 105.127, all *p* < 0.001). Crucially, school avoidance (SA) was identified as the second most prominent independent associate, demonstrating a similarly powerful risk elevation effect (Boys: OR = 1.427, 95% CI: 1.354–1.503, Wald = 178.545; Girls: OR = 1.431, 95% CI: 1.354–1.511, Wald = 164.484, all *p* < 0.001). Other anxiety domains, such as panic disorder (PD) and separation anxiety (SepA), remained statistically significant correlates but presented lower standardized odds ratios(see Table S2). Multicollinearity diagnostics performed across the pooled, male-specific, and female-specific cohorts indicated that the Variance Inflation Factors (VIF) for all five anxiety predictors were consistently below 5.0, proving that the parameter estimations within the multivariate regression models were robustly stable and entirely free from multi-collinearity-driven distortion.

Furthermore, to address potential developmental shifts in the symptom-level relationship, we examined age as a potential moderator using hierarchical binary logistic regression. While anxiety remained a potent and significant correlate of behavioral problem status (B = 0.118, Exp(B) = 1.126, *p* < 0.001), the interaction between anxiety and age was not statistically significant (B = 0.003, *p* = 0.320). These results indicate that the strength of the association between anxiety and the risk of manifesting behavioral problems is stable across the 9–16 age range in this sample, and does not show significant moderation by developmental stage.

### Network structure and centrality measure analysis

**Fig. 2:**
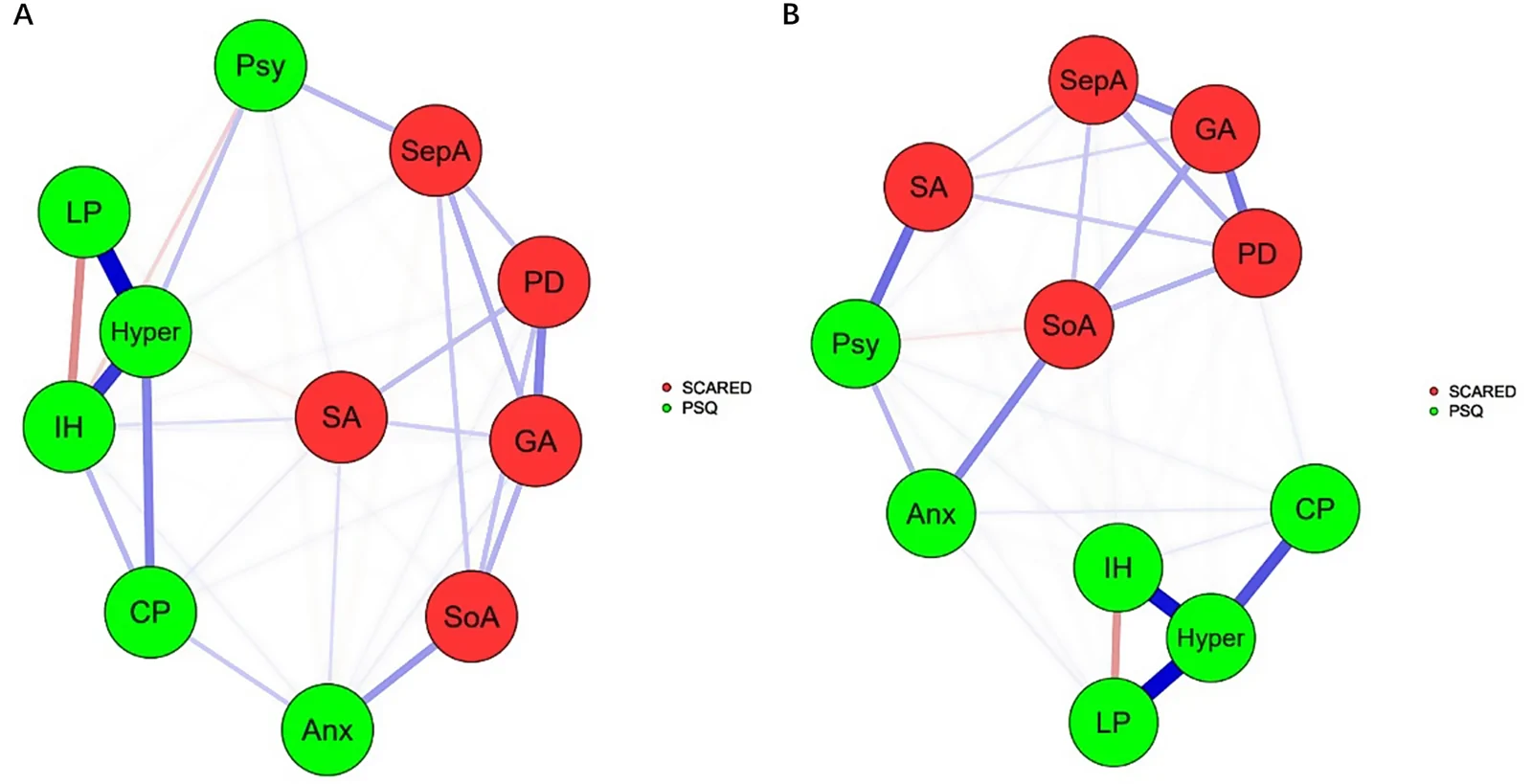
Gender-specific network structures of anxiety and behavioral problems. Notes: (**A**) Network structure for boys showing 11 nodes connected by 43edges (78.18% edge density; sparsity = 21.82%). (**B**) Network structure for girls with 11 nodes connected by 49 edges (89.09% edge density; sparsity = 10.91%)

The anxiety-problem behavior network models for boys and girls are shown in Fig. 2. Panel A depicts the anxiety-problem behavior network for boys, consisting of 11 nodes, with 43 non-zero edges, accounting for 78.18% of the total edges, and a sparsity of 21.82%. Panel B represents the anxiety-problem behavior network for girls, also comprising 11 nodes, with 49 non-zero edges, accounting for 89.09% of the total edges, and a sparsity of 10.91%. While the girls’ network displayed a higher breadth of interconnectedness (greater edge density), the significantly higher global strength observed in boys reflects a greater absolute magnitude and intensity among localized edge connections. The correlation matrices are shown in Table S3 and S4.

**Fig. 3:**
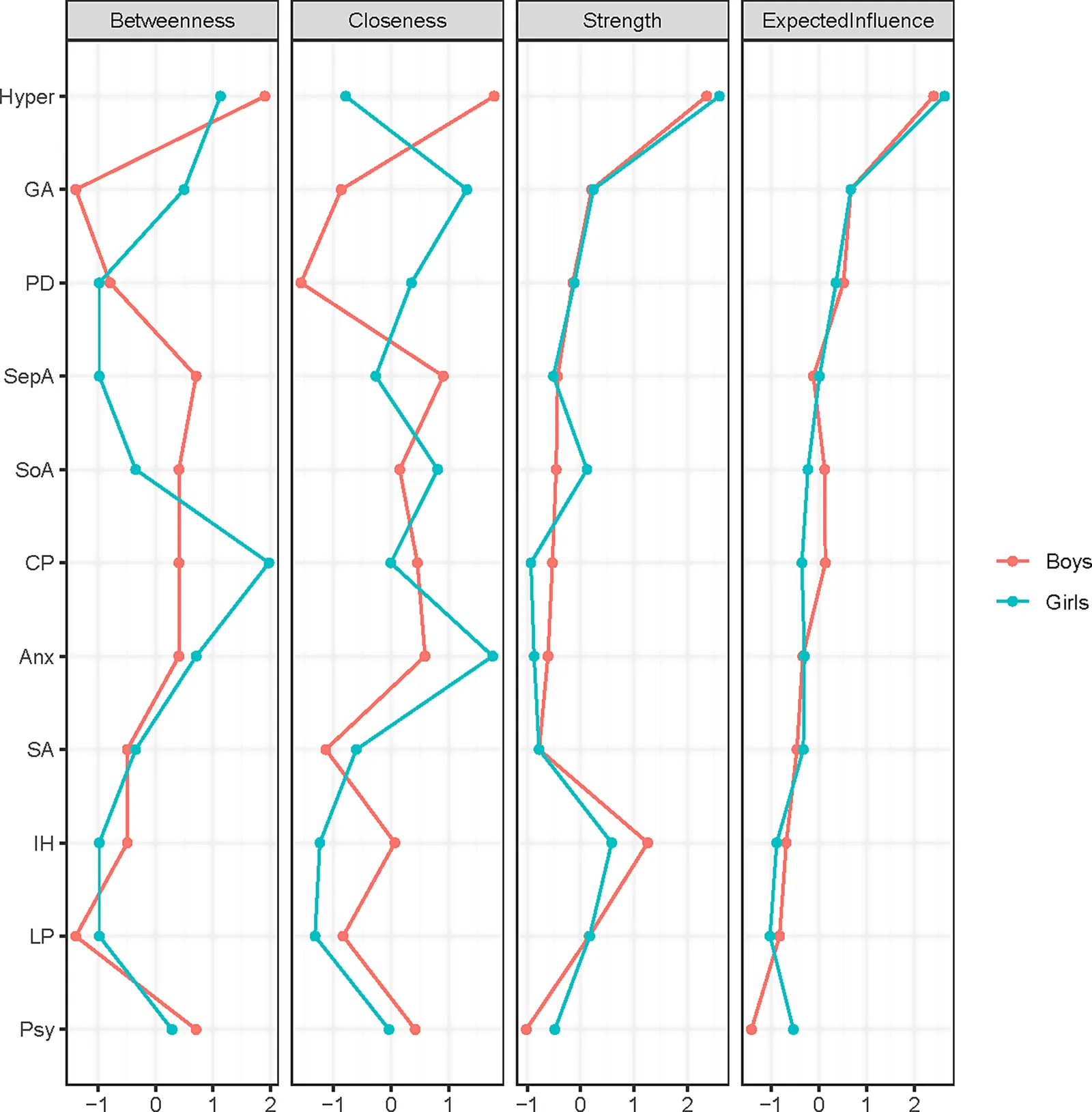
Centrality measures of anxiety and behavioral problem networks by gender

After constructing the network, we calculated several centrality measures to identify key nodes within the network (Fig. 3, Table S5, and S6). In Fig. 3, red indicates boys, and blue indicates girls. It can be observed that, regardless of gender, Hyper and GA are the two dimensional domains with the highest Expected Influence in both the boys’ and girls’ anxiety-problem behavior networks. This suggests that Hyper and GA have the greatest influence on other trait domains within the network, potentially playing significant roles in the propagation of influence across the network.

To safeguard our centrality interpretations against potential psychometric distortions driven by item overlap within the PSQ scale (specifically regarding the Hyperkinesis Index, which shares constituent items with LP and IH), a rigorous sensitivity analysis was executed by re-estimating the age-residualized network after completely excluding the Hyperkinesis Index (see Supplementary Figures S1 and Tables S7–S10). The resulting 10-node network topology demonstrated exceptional structural resilience. Although the removal of the overlapping index naturally shifted the absolute Expected Influence rankings—positioning Conduct Problems (CP; Boys = 1.681, Girls = 1.675) and Generalized Anxiety (GA; Boys = 1.014, Girls = 1.565) as the new primary internalizing and externalizing hubs—the regularized edge weights among the remaining 10 distinct domains remained virtually un-changed. This stable cross-model alignment confirms that while the absolute centrality of Hyperkinesis is partially amplified by scale construction, it does not distort the broader psychopathological network architecture.

These comparative results, visualized in Fig. 2, underscore that while some core domains are shared, the functional routes linking internalizing distress to externalizing manifestations are distinct and more densely integrated in boys.

### Bridge domains and bridge connections

In the anxiety-problem behavior network structure for boys, the primary bridging domains were Anxiety (Anx, bridge EI = 0.494) and Social anxiety (SoA, bridge EI = 0.307). Their connections were primarily sustained through three robust bridge pathways: Anxiety and Social Anxiety (Anx-SoA, edge weight = 0.286), Separation Anxiety and Psychosomatic problem (SepA-Psy, edge weight = 0.198) and School Avoidance and Anxiety (SA-Anx, edge weight = 0.111) (Figure S2).

In contrast, the girls’ network was predominantly bridged by School Avoidance (SA, bridge EI = 0.413) and Anxiety (Anx, bridge EI = 0.391). The pathways connecting School Avoidance and Psychosomatic problem (SA-Psy, edge weight = 0.352) and Anxiety and Social Anxiety (Anx-SoA, edge weight = 0.295) were the most prominent (Figure S3). Notably, the SA-Psy edge weight in girls (0.352) was substantially higher than that in boys (0.075), representing the most pronounced weight difference across all bridge-related connections (E = 0.277, *p* < 0.001) (Table S11).

### Network stability and accuracy

Network stability and accuracy were evaluated using non-parametric and case-dropping bootstrap procedures (1,000 iterations). As shown in Figure S4, the bootstrap mean edge weights closely aligned with the original sample edge strengths, indicating high accuracy in edge estimation for both gender networks. Regarding centrality stability, the Correlation Stability (CS) coefficient for Expected Influence was 0.75 for both the boys’ and girls’ networks, significantly exceeding the preferred threshold of 0.50. Furthermore, the stability of bridge expected influence was equally robust, with a CS-coefficient of 0.75 for both groups (Figure S5). These results demonstrate that the network structures and node rankings are highly stable and provide a reliable foundation for subsequent comparative analyses.

### Network comparison between boys group and girls group

The Network Comparison Test (NCT) revealed significant differences in the overall structure and global strength of the networks between boys and girls (Fig. 2). Specifically, the network structural invariance test showed a significant difference in the overall network structure between boys and girls (M = 0.277, *p* < 0.001). Additionally, the global strength invariance test indicated a significant difference in the global strength of the networks between boys and girls (S = 0.303, *p* < 0.001), with the global strength of the boys’ network (5.881) being slightly higher than that of the girls’ network (5.578, Figure S6).

The edge invariance test further compared whether there were significant differences in the edge weights between the boys and girls networks, i.e., whether the connection strengths between certain nodes differed significantly between the sexes. Table S11 lists the connections where significant weight differences were observed between the boys and girls networks. When ranked by the magnitude of difference (Test Statistic E), the most pronounced gender-specific pathways included SA-Psy (Girls = 0.352, Boys = 0.075, E = 0.277, *p* < 0.001) and Psy-Anx (Girls = 0.177, Boys = -0.043, E = 0.220, *p* < 0.001). Notably, boys showed significantly stronger associations in externalizing-related paths, such as CP-IH (Boys = 0.200, Girls = 0.080, E = 0.120) and LP-Hyper (Boys = 0.712, Girls = 0.619, E = 0.092) compared to girls (*p* < 0.001).

## Discussion

The current study reveals a close positive relationship between anxiety and behavioral problems in children. Furthermore, anxiety, particularly generalized anxiety (GA) followed by school avoidance (SA), shows the most robust independent associations with behavioral problems. A notable finding is that girls exhibit significantly higher levels of anxiety, whereas boys show significantly more prominent behavioral problems. The anxiety-behavioral problem networks of boys and girls display significant differences in their overall structure and global strength. Despite these structural distinctions, Hyperkinesis (Hyper) and Generalized Anxiety (GA) emerged as core domains for both genders.

### The relationship between anxiety and behavioral problems

This study confirms the significant association between anxiety and behavioral problems, consistent with extensive prior research. This association remains robust even after partialling out the effect of chronological age, underscoring a developmental stability in the internalizing-externalizing interplay. Anxiety, a prevalent mental health condition in children and adolescents, is linked to a broad spectrum of behavioral problems, including aggressive behaviors [37–41], negative peer interactions [42], peer rejection [43], social dysfunction [44, 45], and impaired academic functioning [44, 46]. These connections are likely mediated by complex cognitive processing and social environmental influences. Dual-process theory suggests anxiety can promote unethical conduct by shifting cognitive processing from deliberate to intuitive modes, leading individuals to prioritize self-interest over moral standards [47]. Children experiencing anxiety symptoms are often more prone to withdrawing from social engagement, which can limit their opportunities to practice and develop social skills, potentially resulting in diminished social competence [48]. Conversely, children who participate in social skills training programs are less likely to experience various forms of anxiety [42, 49]. Additionally, external factors such as parental stress and a lack of emotional support can exacerbate behavioral problems in anxious adolescents. Children with severe anxiety and a history of adverse childhood experiences are particularly susceptible to exhibiting behavioral problems, highlighting the critical role of environmental factors in the trajectory of behavioral problems development [6].

Crucially, the complex interplay of psychological, environmental, and family factors suggests that children’s behavioral problems can, in turn, further exacerbate their anxiety. This indicates a dynamic feedback loop rather than a simple unidirectional cause-effect relationship. For instance, extensive research demonstrates that peer victimization [50, 51] and aggression [52] significantly contribute to an intensification of social anxiety symptoms in adolescents. Children with heightened punishment sensitivity may be more predisposed to anxiety because they are more likely to perceive threats, leading to defensive behaviors that can manifest as overt behavioral problems. Conversely, a high reward sensitivity, when unfulfilled, can result in frustration and behavioral issues, which then further intensify anxiety [53]. Children struggling with behavioral problems frequently experience dissatisfaction with school due to conflicts with peers and unmet educational needs. This dissatisfaction can spiral into increased self-blame and heightened anxiety as they contend with the challenges of navigating their social and academic environments [54]. Similarly, children with learning difficulties often present with co-occurring anxiety and behavioral problems. These issues are profoundly influenced by parenting styles and the prevailing family atmosphere, which can compound both anxiety and behavioral problems [55]. This bidirectional relationship highlights that school-based mental health screenings should monitor both domains simultaneously. Early preventive efforts that address mild behavioral disruptions may help mitigate the escalation of internalizing anxiety, and vice versa, underscoring the need for integrated, multi-faceted supportive strategies in school settings.

A salient finding of this study is that after neutralizing scale variance through Z-score standardization, generalized anxiety (GA) emerged as the single strongest independent correlate of behavioral problems, which perfectly converges with our topological network findings identifying GA as the premier psychopathological hub (highest Expected Influence). Crucially, school avoidance (SA) was identified as the second most prominent independent associate in both boys and girls (Table S2), demonstrating a powerful risk elevation effect. This particular observation suggests that identifying and addressing both generalized cognitive worry and school-related avoidance early could yield a disproportionately large positive impact on a child’s overall behavioral health. This points to critical focal points for early school-based support. It is important to note that the relationship between school avoidance and behavioral problems is likely bidirectional; while school-related anxiety can manifest as behavioral issues, existing behavioral problems may also lead to school-based social exclusion, thereby reinforcing avoidance patterns. Research by Yong and Abdullah underscores the significant role of self-regulation skills and parenting styles in influencing both school readiness and the manifestation of behavioral problems [56]. Students who demonstrate poor self-management and lower levels of school readiness typically exhibit a greater number of behavioral problems. This indicates that anxieties related to school performance and readiness can contribute to behavioral problems, as children may feel overwhelmed and inadequately equipped to meet academic demands [56]. Severe school avoidance can lead to truancy; a survey involving 4500 American children and adolescents revealed that 17.5% of children with anxious school refusal struggled to form friendships due to aggression, a proportion considerably higher than among children who simply truanted [57]. Furthermore, the perceived quality of school life significantly predicts student involvement in bullying and victimization. Students holding a negative perception of their school environment are more prone to participating in or becoming victims of bullying, which can act as both a precursor and a consequence of school-related fears [58].

### Gender differences in anxiety and behavioral problems

The results of the z-test demonstrate that girls scored significantly higher than boys across all types of anxiety(Table 1). Conversely, boys exhibited significantly higher scores on conduct problems (CP), learning problems (LP), impulsive-hyperactivity (IH), and the hyperkinesis index (Hyper) (Table 1). This finding indicates that girls experience more severe anxiety, while boys present with more pronounced behavioral problems. These results align with existing literature, reporting higher test anxiety in primary school girls [59], and greater social anxiety prevalence among adolescent girls [60–62]. Observed gender differences in internalizing problem trajectories suggest girls are more likely to experience increased anxiety symptoms over time, while boys’ symptoms may stabilize or decrease [63]. Regarding behavioral problems, evidence suggests that girls typically display more indirect aggression than boys, while boys tend to exhibit more physical aggression [64, 65]. Biological and sociological factors contribute to differential responses to peer provocation between boys and girls, with males often presenting patterns more consistent with externalizing problems such as aggression [66]. Indeed, males constitute the majority in both childhood-onset and adolescent-onset conduct disorders [67].

The observed disparities in anxiety and behavioral patterns persist in our network models after residualizing for age. However, because our study relies entirely on behavioral screening scales, these architectural configurations must be interpreted with extreme caution and cannot empirically disentangle biological explanations from social gender roles. From a highly speculative developmental psychopathology perspective, literature-based physiological profiles suggest that sex-differentiated trajectories may tentatively relate to variations in neuroendocrine markers. For instance, androgens and testosterone have been investigated as concurrent correlates of physical and indirect aggression profiles in childhood longitudinal studies, theoretically aligning with the earlier observation of overt behavioral disruptions in males [68–70]. Concurrently, post-pubertal variance in internalizing features has been tentatively linked in translational neuroimaging literature to developmental variations in fronto-amygdala structural connectivity, which may manifest as distinct anxiety-expression profiles across sexes, though our behavioral screening design cannot formally test these somatic trajectories [71]. Cognitively, girls are more prone to employing cognitive avoidance as a coping strategy, a pattern consistent with findings in adult anxiety research. This avoidance behavior, when compared to boys’ more frequent use of problem-solving strategies, may contribute to higher anxiety levels in girls [72]. Understanding these mechanisms is crucial for designing gender-sensitive preventive screening and guidance; for example, school-based emotional support for girls might emphasize adaptive coping, while proactive behavioral guidance for boys might focus on constructive problem-solving.

Second, distinctions in socio-emotional development and temperament contribute to gender disparities. Boys and girls exhibit significant differences in emotional expression. A meta-analysis revealed that girls tend to display more internalizing emotions, such as anxiety, whereas boys more frequently express externalizing emotions, like anger. When emotions such as fear or empathy are excluded, the presence of certain stressors can lead to behavioral problems, including aggressive conduct and disregard for behavioral rules [9, 10]. Another meta-analysis on temperament-related gender differences found that boys generally demonstrate greater impulsivity than girls, characterized by higher levels of impulsivity, activity, and intense pleasure-seeking [73]. Clear gender differences are also evident in coping strategies for specific problems: girls more often employ emotional coping strategies and rumination, while boys tend to use distraction and aggressive strategies [74, 75]. These emotional gender differences may emerge as early as the preschool years; prior research indicates that during play, preschool girls exhibit more submissive emotions than boys, who tend to express more discordant emotions [76]. Furthermore, compared to boys, girls typically show higher levels of effortful control [73], possess stronger social sensitivity and emotional regulation abilities [77, 78], and exhibit superior language skills [79]. These traits may enhance girls’ adaptability to problems, leading to fewer externalizing behavioral issues, with their difficulties more often manifesting internally. Beyond individual coping strategies, gender-specific socialization and societal expectations play a critical role in shaping symptom expression. From an early age, children are socialized into gender-congruent roles where boys are often encouraged to adhere to norms of ‘toughness’ and emotional stoicism, while girls are granted more latitude to express vulnerability and fear [10]. These social expectations may lead boys to suppress internalizing distress, which subsequently manifests as ‘masculine’ expressions of psychopathology, such as externalizing aggression or conduct problems—a phenomenon where behavioral disruptions act as a proxy for underlying emotional turmoil [9, 11]. Conversely, gender norms that reinforce interpersonal sensitivity and emotional expression in girls may amplify the pathway from generalized anxiety to internalizing symptoms [12].

Furthermore, it is essential to situate these findings within the Chinese cultural context, where gendered socialization and cultural reporting filters operate as highly plausible interpretive frameworks rather than empirically verified variables in our dataset. In traditional Chinese culture, parents and teachers may exhibit differential sensitivity to symptoms based on gendered expectations [80]. For instance, ‘quietness’ and ‘compliance’ are often viewed as ideal feminine traits in Chinese society, which might lead guardians to overlook internalizing anxiety in girls, interpreting it as a personality characteristic rather than a clinical symptom [81]. In contrast, hyperkinesis and conduct problems in boys are more likely to be perceived as threats to social harmony and classroom discipline, leading to higher reporting rates and more proactive help-seeking behaviors for externalizing issues in males [82]. Such diagnostic bias implies that the stronger link between anxiety and behavioral problems observed in boys may reflect, in part, a cultural tendency to attend to the most visible ‘troublesome’ symptoms in males while underreporting the nuanced emotional distress in females. Such localized diagnostic and reporting variations imply that the distinct network clustering and predictive paths documented in our cohort may, in part, reflect these unmeasured cultural and contextual filters alongside potential biological variance. Because our research did not directly measure cultural parameters, parental beliefs, teacher expectations, or help-seeking stigma, the observed sex-differentiated patterns cannot be used to definitive disentangle biological mechanisms from social explanations, necessitating a cautious and tentative interpretation. Acknowledging these speculative socio-cultural lenses is vital for contextualizing youth psychopathology in non-Western populations without overextending the empirical boundaries of static screening data.

### Gender differences in anxiety-behavioral problems networks

Through the application of network analysis, the current study elucidates significant structural differences in the anxiety-behavioral problem networks between boys and girls(Fig. 2, Figure S6). In direct alignment with our reframed second hypothesis (H2), the empirical results confirmed that the two cohorts are driven by highly distinct topological configurations. When interpreting these cross-scale interconnections, a critical psychometric distinction must be maintained regarding the nature of the edges. Specifically, the connections linking self-reported SCARED domains to the parent-rated PSQ-Anxiety and Psychosomatic clusters should be interpreted as robust indicators of cross-informant convergent validity regarding the child’s internalizing distress, rather than a causal cascade from emotion to externalizing misconduct. In contrast, the direct paths connecting anxiety domains to true externalizing clusters—such as Conduct Problems (CP) or Impulsive-Hyperactivity (IH)—provide genuine evidence of cross-domain psychopathological coupling that transcends informant-source variance.

Notably, after controlling for age, this study found that the global strength in the boys’ network (5.881) was significantly higher than that in the girls’ network (5.578), thereby shedding light on the complex architectural differences across genders. This unexpected direction primarily stems from the exceptionally high coupling within the externalizing symptom dimensions among boys (e.g., the LP-Hyper edge weight reaching 0.712). While girls present with widespread, diffuse symptom generalization (higher edge density of 89.09%), boys exhibit a highly condensed, intense, and self-reinforcing externalizing cluster. This pattern suggests that girls may predominantly exhibit more internalizing and emotional behaviors. This could lead to their struggles being harder to detect or attribute solely to external behaviors, potentially resulting in emotional distress in girls being overlooked during routine school-based universal screenings. Similar gender differences have been observed in adult populations; for instance, a large-sample study found that the correlation between anxiety and physical and verbal aggression was significantly higher in males than in females [83]. Additionally, gender differences in the impact of peer pressure on children may contribute to these network distinctions. Boys often experience substantial peer pressure related to misconduct and social aggression, and this pressure can exacerbate behavioral problems, leading to increased anxiety and behavioral challenges [84]. Boys generally face greater pressure regarding peer involvement compared to girls [84].

Despite the observed structural variations between genders, a critical finding is that Hyperkinesis (Hyper) and Generalized Anxiety (GA) emerged as core domains within the anxiety-behavioral problem networks for both boys and girls (Fig. 3). This indicates that while the overall architecture and interplay of symptoms differ significantly by gender, these specific symptoms remain highly influential and central to the psychopathology in both groups. Network analyses of anxiety symptoms in 4964 children and adolescents have similarly demonstrated that GA and panic symptoms consistently remain core domains, irrespective of age or gender [15]. Generalized Anxiety (GA) is characterized by excessive and often uncontrollable worry and tension across multiple domains, such as academic performance, future prospects, and interpersonal relationships. This pervasive worry can significantly impact various aspects of a child’s life, including their academic, family, and social environments [85]. Prior research indicates that individuals with Generalized Anxiety Disorder (GAD) often exhibit a cognitive bias towards worry-related information in their surroundings and tend to interpret ambiguous information as threatening [86, 87]. GA may also have a bidirectional relationship with other anxiety symptoms, such as separation anxiety, school avoidance, and social anxiety, meaning it can both result from and precipitate these other symptoms. Resting-state functional magnetic resonance imaging studies of patients with GAD and Social Anxiety Disorder (SAD) have shown that both conditions exhibit abnormally elevated connectivity between the left angular gyrus and the right insula [88].

Hyperkinesis, characterized by high energy, difficulty with control, impulsivity, and attention deficits, is a behavioral trait that demonstrates robust, direct co-occurrence with behavioral problems and displays strong negative correlations with learning, emotional regulation, and social interaction. Notably, in research related to Attention-Deficit/Hyperactivity Disorder (ADHD), hyperkinesis is considered a primary symptom contributing to academic difficulties and maladjustment in social settings [88, 89]. Hyperkinetic behaviors can be reinforced by environmental factors; for example, academic failures or conflicts with teachers may further exacerbate the expression of hyperkinesis, creating a self-perpetuating cycle [90]. The identification of Hyperkinesis and Generalized Anxiety as core domains, despite gender-specific network structures, suggests a dual-layered screening strategy: these domains should be prioritized as core indicators in universal preventive screenings for all children, while acknowledging the need for gender-specific pathways in early emotional and behavioral support.

However, a psychometric caveat must be acknowledged regarding the exceptionally high centrality of Hyperkinesis. As revealed by our sensitivity analysis, the prominence of the Hyperkinesis Index is partly artifactual, driven by its structural item overlap with the LP and IH domains. Therefore, its position as a core network hub should be interpreted with caution. Rather than a purely unique psychopathological entity, the Hyperkinesis Index operates as a robust, aggregate screening indicator that captures shared variance across multiple behavioral problems. This dual nature—being a psychometric composite yet clinically central—reaffirms its practical utility for large-scale school screenings, while micro-level symptom interactions are better captured by non-overlapping subscales like CP and GA.

### Limitations and future directions

Although this study provides important evidence for gender differences, several limitations exist. First, the cross-sectional design prevents establishing causal relationships. Second, due to strict de-identification protocols, specific identifiers for schools, classrooms, teacher practices, academic performance, or socioeconomic backgrounds were unavailable, preventing multilevel modeling or cluster-robust statistical adjustments to control for institutional nesting dependencies. Third, while nested model comparisons supported strong invariance, the baseline configural model for the SCARED scale yielded a suboptimal absolute fit (CFI = 0.868), meaning cross-sex comparisons require psychometric caution. Fourth, our nodes relied on aggregate domain subscale scores rather than individual symptoms; while this minimized mathematical noise, it inherently obscured fine-grained, within-domain symptom relations. Fifth, several brief subscales demonstrated limited reliability with alphas slightly below 0.70. Sixth, the study utilized a multi-informant design, combining child self-reports (SCARED) with parent ratings (PSQ). While this simultaneously guards against common-method bias, it introduces informant-source variance. The identified bridge pathways between internalizing distress and externalizing manifestations may be partially shaped by informant visibility and parental awareness, thereby artificially inflating or deflating specific cross-informant edge weights. Seventh, certain high-magnitude network edges involving the Hyperkinesis Index are partially driven by scale construction item-overlap artifacts across the PSQ, rather than substantive clinical co-occurrence. Eighth, our statistical configurations lacked critical confounding covariates, including family environments, socioeconomic backgrounds, academic performance, and school-context parameters. Ninth, although linear developmental variance was residualized, potential non-linear age trajectories across the 9–16 range cannot be entirely discounted. Last, this non-clinical community sample lacks formal clinical diagnoses, meaning these dimensional screen indicators may not directly generalize to severe psychiatric cohorts.

Future research, particularly longitudinal network studies, is needed to track the development of these symptom networks over time and to determine whether domains like ‘School avoidance’ and ‘Hyperkinesis’ act as early risk indicators that propagate distress to other domains over time. Additionally, future studies could explore how school-based preventive supportive strategies specifically impact the structure of these networks, thereby providing further validation of our identified core nodes and key screening indicators.

## Conclusion

This study apply network analysis to a large, population-based sample of children to examine the architecture of anxiety and behavioral problems, revealing a highly interconnected network with significant gender-specific manifestations. Our findings are highly consistent with a network-oriented, dimensional perspective on co-occurring internalizing and externalizing domains, demonstrating that these constructs form a complex web of cross-informant covariance without implying directional causality. Collectively, these findings provide critical empirical benchmarks for optimizing early, gender-sensitive mental health screening protocols and administrative risk-identification frameworks within continuous large-scale educational settings.

## Supplementary Information

Below is the link to the electronic supplementary material.

Supplementary Material 1

## Data Availability

The data that support the findings of this study are available from the corresponding author upon reasonable request.

## Abbreviations

SCARED: Screen for Child Anxiety-Related Emotional Disorders
PD: Panic Disorder or Significant Somatic Symptoms
GA: Generalized Anxiety
SepA: Separation Anxiety
SoA: Social Anxiety
SA: School Avoidance
PSQ: Conners Parent Symptom Questionnaire
CP: Conduct problem
LP: Learning problem
Psy: Psychosomatic problem
IH: Impulsive-hyperactive
Anx: Anxiety
Hyper: Hyperkinesis Index
