# Supplementary material for: Gender differences in the association between anxiety and behavioral problems in 31,400 Chinese children: a cross-sectional regression and network analysis
Source: BMC Psychiatry. 2026 Jul 14;26:768. doi: 10.1186/s12888-026-08371-9 (PMC13651017; doi:10.1186/s12888-026-08371-9)
Supplement: Supplementary file 1 [file 12888_2026_8371_MOESM1_ESM.docx]

| Table S1 Summary of Multi-Group Confirmatory Factor Analysis and Measurement Invariance Across Sexes (MLR Estimator) | | | | | | | | | | | | |
| --- | --- | --- | --- | --- | --- | --- | --- | --- | --- | --- | --- | --- |
| **Scale and Model** | **χ2** | **df** | **CFI** | **TLI** | **RMSEA** | **SRMR** | **Δχ2** | **Δdf** | **p(Δχ2)** | **ΔCFI** | **ΔTLI** | **ΔRMSEA** |
| **SCARED** |  |  |  |  |  |  |  |  |  |  |  |  |
| M1: Configural Invariance | 30874.19 | 1538 | 0.868 | 0.859 | 0.046 | 0.041 | - | - | - | - | - | - |
| M2: Metric Invariance | 30914.81 | 1574 | 0.867 | 0.861 | 0.046 | 0.043 | 248.17 | 36 | <0.001 | -0.001 | 0.002 | 0.000 |
| M3: Scalar Invariance | 31817.78 | 1610 | 0.864 | 0.861 | 0.046 | 0.043 | 1048.69 | 36 | <0.001 | -0.003 | 0.000 | 0.000 |
| **PSQ** |  |  |  |  |  |  |  |  |  |  |  |  |
| M1: Configural Invariance | 15538.21 | 734 | 0.896 | 0.885 | 0.051 | 0.043 | - | - | - | - | - | - |
| M2: Metric Invariance | 15803.95 | 758 | 0.892 | 0.885 | 0.051 | 0.048 | 344.94 | 24 | <0.001 | -0.003 | 0.000 | 0.000 |
| M3: Scalar Invariance | 16708.42 | 782 | 0.888 | 0.884 | 0.052 | 0.048 | 1327.39 | 24 | <0.001 | -0.004 | -0.001 | 0.000 |

| Table S2 Multivariate Logistic Regression Analysis Results for Boys and Girls | | | | | | | |
| --- | --- | --- | --- | --- | --- | --- | --- |
| Sex | Predictor | Estimate (B) | Standard Error | Wald χ2 | p | OR | 95% CI for OR |
| Boy | Intercept | -2.391 | 0.418 | 32.670 | ＜0.001 | 0.092 | - |
|  | Age(years) | 0.023 | 0.039 | 0.341 | 0.559 | 1.023 | [0.948, 1.103] |
|  | PD | 0.316 | 0.035 | 83.365 | ＜0.001 | 1.372 | [1.282, 1.469] |
|  | GA | 0.404 | 0.037 | 118.524 | ＜0.001 | 1.498 | [1.393, 1.611] |
|  | SepA | 0.226 | 0.031 | 52.217 | ＜0.001 | 1.254 | [1.179, 1.334] |
|  | SoA | 0.215 | 0.030 | 50.597 | ＜0.001 | 1.240 | [1.169, 1.316] |
|  | SA | 0.356 | 0.027 | 178.545 | ＜0.001 | 1.427 | [1.354, 1.503] |
| Girl | Intercept | -2.713 | 0.475 | 32.648 | ＜0.001 | 0.066 | - |
|  | Age(years) | 0.012 | 0.044 | 0.078 | 0.780 | 1.012 | [0.929, 1.104] |
|  | PD | 0.274 | 0.036 | 58.767 | ＜0.001 | 1.315 | [1.226, 1.411] |
|  | GA | 0.390 | 0.038 | 105.127 | ＜0.001 | 1.477 | [1.371, 1.591] |
|  | SepA | 0.254 | 0.033 | 58.763 | ＜0.001 | 1.290 | [1.209, 1.377] |
|  | SoA | 0.298 | 0.034 | 78.336 | ＜0.001 | 1.347 | [1.261, 1.439] |
|  | SA | 0.358 | 0.028 | 164.484 | ＜0.001 | 1.431 | [1.354, 1.511] |

| Table S3 The correlation matrix of boys group | | | | | | | | | | | | | | | | | | | | | |
| --- | --- | --- | --- | --- | --- | --- | --- | --- | --- | --- | --- | --- | --- | --- | --- | --- | --- | --- | --- | --- | --- |
|  | PD | | GA | | | SepA | | SoA | SA | CP | | | LP | | Psy | IH | | Anx | | | Hyper |
| PD | 0 | | 0.337 | | | 0.178 | | 0.177 | 0.183 | 0.027 | | | 0 | | 0 | 0.050 | | 0.063 | | | 0 |
| GA | 0.337 | | 0 | | | 0.218 | | 0.214 | 0.143 | 0.062 | | | 0.010 | | -0.034 | 0.049 | | 0.077 | | | 0 |
| SepA | 0.178 | | 0.218 | | | 0 | | 0.160 | 0 | -0.006 | | | 0.019 | | 0.198 | -0.025 | | -0.043 | | | 0.064 |
| SoA | 0.177 | | 0.214 | | | 0.160 | | 0 | 0 | 0.000 | | | 0.043 | | -0.012 | -0.009 | | 0.286 | | | 0 |
| SA | 0.183 | | 0.143 | | | 0 | | 0 | 0 | 0.083 | | | 0 | | 0.075 | 0.110 | | 0.111 | | | -0.080 |
| CP | 0.027 | | 0.062 | | | -0.006 | | 0.000 | 0.083 | 0 | | | 0 | | 0 | 0.200 | | 0.154 | | | 0.343 |
| LP | 0 | | 0.010 | | | 0.019 | | 0.043 | 0 | 0 | | | 0 | | 0.020 | -0.323 | | 0 | | | 0.712 |
| Psy | 0 | | -0.034 | | | 0.198 | | -0.012 | 0.075 | 0 | | | 0.020 | | 0 | -0.137 | | -0.043 | | | 0.178 |
| IH | 0.050 | | 0.049 | | | -0.025 | | -0.009 | 0.110 | 0.200 | | | -0.323 | | -0.137 | 0 | | 0.070 | | | 0.552 |
| Anx | 0.063 | | 0.077 | | | -0.043 | | 0.286 | 0.111 | 0.154 | | | 0 | | -0.043 | 0.070 | | 0 | | | 0 |
| Hyper | 0 | | 0 | | | 0.064 | | 0 | -0.080 | 0.343 | | | 0.712 | | 0.178 | 0.552 | | 0 | | | 0 |
| Table S4 The correlation matrix of girls group | | | | | | | | | | | | | | | | | | | | | |
|  | | PD | | GA | SepA | | SoA | | SA | | CP | LP | | Psy | | | IH | | Anx | Hyper | |
| PD | | 0 | | 0.323 | 0.204 | | 0.185 | | 0.136 | | 0.019 | 0.000 | | 0.039 | | | 0.030 | | 0.029 | 0.017 | |
| GA | | 0.323 | | 0 | 0.266 | | 0.234 | | 0.104 | | 0.071 | 0.000 | | 0.000 | | | 0.018 | | 0.034 | 0.030 | |
| SepA | | 0.204 | | 0.266 | 0 | | 0.145 | | 0.112 | | 0.012 | 0 | | 0.053 | | | 0.047 | | 0.025 | 0.008 | |
| SoA | | 0.185 | | 0.234 | 0.145 | | 0 | | 0 | | 0.007 | 0.026 | | -0.091 | | | -0.035 | | 0.295 | 0 | |
| SA | | 0.136 | | 0.104 | 0.112 | | 0 | | 0 | | 0.000 | 0.032 | | 0.352 | | | 0.037 | | 0 | 0 | |
| CP | | 0.019 | | 0.071 | 0.012 | | 0.007 | | 0.000 | | 0 | 0.007 | | 0.061 | | | 0.080 | | 0.079 | 0.417 | |
| LP | | 0 | | 0.000 | 0 | | 0.026 | | 0.032 | | 0.007 | 0 | | 0 | | | -0.259 | | 0.069 | 0.619 | |
| Psy | | 0.039 | | 0.000 | 0.053 | | -0.091 | | 0.352 | | 0.061 | 0 | | 0 | | | 0 | | 0.177 | 0.000 | |
| IH | | 0.030 | | 0.018 | 0.047 | | -0.035 | | 0.037 | | 0.080 | -0.259 | | 0 | | | 0 | | 0 | 0.577 | |
| Anx | | 0.029 | | 0.034 | 0.025 | | 0.295 | | 0 | | 0.079 | 0.069 | | 0.177 | | | 0 | | 0 | 0.023 | |
| Hyper | | 0.017 | | 0.030 | 0.008 | | 0 | | 0 | | 0.417 | 0.619 | | 0.000 | | | 0.577 | | 0.023 | 0 | |

| Table S5 The node centrality index of boys group | | | | |
| --- | --- | --- | --- | --- |
| node | Betweenness | Closeness | Strength | ExpectedInfluence |
| PD | -0.786 | -1.566 | -0.147 | 0.515 |
| GA | -1.382 | -0.865 | 0.207 | 0.669 |
| SepA | 0.705 | 0.911 | -0.434 | -0.115 |
| SoA | 0.406 | 0.151 | -0.461 | 0.120 |
| SA | -0.488 | -1.134 | -0.780 | -0.461 |
| CP | 0.406 | 0.460 | -0.531 | 0.134 |
| LP | -1.382 | -0.833 | 0.160 | -0.825 |
| Psy | 0.705 | 0.422 | -1.024 | -1.415 |
| IH | -0.488 | 0.068 | 1.260 | -0.684 |
| Anx | 0.406 | 0.592 | -0.615 | -0.338 |
| Hyper | 1.897 | 1.795 | 2.367 | 2.401 |

| Table S6 The node centrality index of girls group | | | | |
| --- | --- | --- | --- | --- |
| node | Betweenness | Closeness | Strength | ExpectedInfluence |
| PD | -0.974 | 0.355 | -0.117 | 0.357 |
| GA | 0.497 | 1.321 | 0.237 | 0.666 |
| SepA | -0.974 | -0.265 | -0.513 | 0.011 |
| SoA | -0.344 | 0.812 | 0.118 | -0.230 |
| SA | -0.344 | -0.604 | -0.783 | -0.326 |
| CP | 1.967 | -0.004 | -0.936 | -0.358 |
| LP | -0.974 | -1.322 | 0.169 | -1.025 |
| Psy | 0.286 | -0.035 | -0.486 | -0.536 |
| IH | -0.974 | -1.238 | 0.582 | -0.885 |
| Anx | 0.707 | 1.769 | -0.876 | -0.305 |
| Hyper | 1.127 | -0.789 | 2.606 | 2.632 |

| Table S7 The correlation matrix of boys group (without Hyper) | | | | | | | | | | |
| --- | --- | --- | --- | --- | --- | --- | --- | --- | --- | --- |
|  | PD | GA | SepA | SoA | SA | CP | LP | Psy | IH | Anx |
| PD | 0 | 0.335 | 0.180 | 0.177 | 0.183 | 0.031 | 0 | 0 | 0.063 | 0.062 |
| GA | 0.335 | 0 | 0.223 | 0.213 | 0.142 | 0.071 | 0.009 | -0.036 | 0.063 | 0.075 |
| SepA | 0.180 | 0.223 | 0 | 0.161 | 0 | 0 | 0.107 | 0.215 | 0 | -0.037 |
| SoA | 0.177 | 0.213 | 0.161 | 0 | -0.001 | 0 | 0.060 | -0.013 | -0.008 | 0.286 |
| SA | 0.183 | 0.142 | 0 | -0.001 | 0 | 0.050 | -0.058 | 0.057 | 0.075 | 0.112 |
| CP | 0.031 | 0.071 | 0 | 0 | 0.050 | 0 | 0.371 | 0.064 | 0.511 | 0.163 |
| LP | 0 | 0.009 | 0.107 | 0.060 | -0.058 | 0.371 | 0 | 0.210 | 0.091 | 0 |
| Psy | 0 | -0.036 | 0.215 | -0.013 | 0.057 | 0.064 | 0.210 | 0 | -0.036 | -0.044 |
| IH | 0.063 | 0.063 | 0 | -0.008 | 0.075 | 0.511 | 0.091 | -0.036 | 0 | 0.084 |
| Anx | 0.062 | 0.075 | -0.037 | 0.286 | 0.112 | 0.163 | 0 | -0.044 | 0.084 | 0 |

| Table S8 The correlation matrix of girls group (without Hyper) | | | | | | | | | | |
| --- | --- | --- | --- | --- | --- | --- | --- | --- | --- | --- |
|  | PD | GA | SepA | SoA | SA | CP | LP | Psy | IH | Anx |
| PD | 0 | 0.325 | 0.204 | 0.187 | 0.135 | 0.030 | 0.005 | 0.040 | 0.052 | 0.030 |
| GA | 0.325 | 0 | 0.266 | 0.235 | 0.102 | 0.088 | 0.029 | 0 | 0.046 | 0.033 |
| SepA | 0.204 | 0.266 | 0 | 0.146 | 0.112 | 0.013 | 0.010 | 0.054 | 0.065 | 0.024 |
| SoA | 0.187 | 0.235 | 0.146 | 0 | 0 | 0.022 | 0.059 | -0.095 | -0.025 | 0.297 |
| SA | 0.135 | 0.102 | 0.112 | 0 | 0 | 0 | 0.027 | 0.352 | 0.031 | 0.006 |
| CP | 0.030 | 0.088 | 0.013 | 0.022 | 0 | 0 | 0.387 | 0.062 | 0.444 | 0.093 |
| LP | 0.005 | 0.029 | 0.010 | 0.059 | 0.027 | 0.387 | 0 | 0.066 | 0.118 | 0.108 |
| Psy | 0.040 | 0 | 0.054 | -0.095 | 0.352 | 0.062 | 0.066 | 0 | 0.075 | 0.179 |
| IH | 0.052 | 0.046 | 0.065 | -0.025 | 0.031 | 0.444 | 0.118 | 0.075 | 0 | 0.061 |
| Anx | 0.030 | 0.033 | 0.024 | 0.297 | 0.006 | 0.093 | 0.108 | 0.179 | 0.061 | 0 |

| Table S9 The node centrality index of boys group (without Hyper) | | | | |
| --- | --- | --- | --- | --- |
| node | Betweenness | Closeness | Strength | ExpectedInfluence |
| PD | -0.034 | -0.092 | 0.509 | 0.754 |
| GA | -1.047 | 0.515 | 1.249 | 1.014 |
| SepA | 0.979 | 0.975 | -0.061 | 0.031 |
| SoA | 0.304 | 1.162 | -0.085 | 0.128 |
| SA | -1.047 | -1.955 | -1.393 | -1.136 |
| CP | 1.655 | 0.016 | 1.756 | 1.681 |
| LP | 0.642 | -0.457 | -0.154 | -0.204 |
| Psy | -1.047 | -0.150 | -1.403 | -1.705 |
| IH | -1.047 | -1.096 | -0.029 | 0.004 |
| Anx | 0.642 | 1.082 | -0.390 | -0.567 |

| Table S10 The node centrality index of girls group (without Hyper) | | | | |
| --- | --- | --- | --- | --- |
| node | Betweenness | Closeness | Strength | ExpectedInfluence |
| PD | -1.135 | 0.452 | 0.458 | 0.754 |
| GA | 0.585 | 1.228 | 1.342 | 1.565 |
| SepA | -1.135 | -0.124 | -0.408 | -0.040 |
| SoA | -0.103 | 0.867 | 0.893 | -0.517 |
| SA | -0.103 | -0.605 | -1.384 | -0.935 |
| CP | 1.616 | -0.816 | 1.461 | 1.675 |
| LP | -1.135 | -1.168 | -1.054 | -0.633 |
| Psy | 0.585 | -0.196 | -0.196 | -1.165 |
| IH | -0.447 | -1.238 | -0.235 | -0.232 |
| Anx | 1.272 | 1.598 | -0.878 | -0.471 |

| Table S11 Detailed Edge Weight Differences Between Boys and Girls | | | | | |
| --- | --- | --- | --- | --- | --- |
| Edge Pair | Boys' Edge Weight | Girls' Edge Weight | p-value | Test Statistic E |  |
| SA-Psy | 0.075 | 0.352 | 0.001 | 0.277 |  |
| Psy-Anx | -0.043 | 0.177 | 0.001 | 0.220 |  |
| Psy-IH | -0.137 | 0.059 | 0.001 | 0.196 |  |
| Psy-Hyper | 0.178 | 0.000 | 0.001 | 0.178 |  |
| SepA-Psy | 0.198 | 0.053 | 0.001 | 0.145 |  |
| CP-IH | 0.200 | 0.080 | 0.001 | 0.120 |  |
| SepA-SA | 0.000 | 0.112 | 0.001 | 0.112 |  |
| SA-Anx | 0.111 | 0.008 | 0.001 | 0.103 |  |
| LP-Hyper | 0.712 | 0.619 | 0.001 | 0.092 |  |
| SA-CP | 0.083 | 0.000 | 0.001 | 0.083 |  |
| SoA-Psy | -0.012 | -0.091 | 0.001 | 0.078 |  |
| CP-Anx | 0.154 | 0.079 | 0.001 | 0.074 |  |
| CP-Hyper | 0.343 | 0.417 | 0.001 | 0.074 |  |
| SA-IH | 0.110 | 0.037 | 0.001 | 0.073 |  |
| SepA-IH | -0.025 | 0.047 | 0.001 | 0.072 |  |
| LP-Anx | 0.000 | 0.069 | 0.001 | 0.069 |  |
| SepA-Anx | -0.043 | 0.025 | 0.001 | 0.068 |  |
| LP-IH | -0.323 | -0.259 | 0.001 | 0.065 |  |
| SA-Hyper | -0.080 | -0.016 | 0.001 | 0.064 |  |
| CP-Psy | 0.000 | 0.061 | 0.001 | 0.061 |  |
| SepA-Hyper | 0.064 | 0.008 | 0.001 | 0.056 |  |
| PD-SA | 0.183 | 0.136 | 0.001 | 0.048 |  |
| GA-SepA | 0.218 | 0.266 | 0.001 | 0.047 |  |
| GA-Anx | 0.077 | 0.034 | 0.001 | 0.043 |  |
| GA-SA | 0.143 | 0.104 | 0.001 | 0.040 |  |
| PD-Psy | 0.000 | 0.039 | 0.001 | 0.039 |  |
| IH-Anx | 0.070 | 0.032 | 0.001 | 0.038 |  |
| PD-Anx | 0.063 | 0.029 | 0.002 | 0.034 |  |
| GA-Psy | -0.034 | 0.000 | 0.001 | 0.034 |  |
| SA-LP | 0.000 | 0.032 | 0.001 | 0.032 |  |
| GA-IH | 0.049 | 0.018 | 0.001 | 0.031 |  |
| GA-Hyper | 0.000 | 0.030 | 0.001 | 0.030 |  |
| SoA-Hyper | 0.000 | 0.029 | 0.001 | 0.029 |  |
| LP-Psy | 0.020 | 0.048 | 0.007 | 0.028 |  |
| PD-SepA | 0.178 | 0.204 | 0.023 | 0.026 |  |
| SoA-IH | -0.009 | -0.035 | 0.001 | 0.026 |  |
| IH-Hyper | 0.552 | 0.577 | 0.002 | 0.025 |  |

Figure S1 Gender-specific network structures of anxiety and behavioral problems (without Hyper)

Notes: (A) Network structure for boys. (B) Network structure for girls.

Figure S2 Bridge Centrality Metrics for Anxiety-Behavior Network in Boys

Figure S3 Bridge Centrality Metrics for Anxiety-Behavior Network in Girls

Figure S4. The edge stability using standard bootstrapping and case bootstrapping methods.

Notes: (A) The edge stability of the boys' network. (B) The edge stability of the girls' network.

Figure S5. Stability of bridge expected influence in anxiety-behavior networks

Notes: (A) The stability of the boys' network. (B) The stability of the girls' network.

Figure S6. Network comparison between boys and girls: structural and global strength differences
